# fMLP-Induced IL-8 Release Is Dependent on NADPH Oxidase in Human Neutrophils

**DOI:** 10.1155/2015/120348

**Published:** 2015-11-08

**Authors:** María A. Hidalgo, María D. Carretta, Stefanie E. Teuber, Cristian Zárate, Leonardo Cárcamo, Ilona I. Concha, Rafael A. Burgos

**Affiliations:** ^1^Laboratory of Molecular Pharmacology, Institute of Pharmacology and Morphophysiology, Faculty of Veterinary Sciences, Universidad Austral de Chile, Independencia 631, 5110566 Valdivia, Chile; ^2^Institute of Biochemistry and Microbiology, Faculty of Sciences, Universidad Austral de Chile, Independencia 631, 5110566 Valdivia, Chile

## Abstract

N-Formyl-methionyl-leucyl-phenylalanine (fMLP) and platelet-activating factor (PAF) induce similar intracellular signalling profiles; but only fMLP induces interleukin-8 (IL-8) release and nicotinamide adenine dinucleotide phosphate reduced (NADPH) oxidase activity in neutrophils. Because the role of ROS on IL-8 release in neutrophils is until now controversial, we assessed if NADPH oxidase is involved in the IL-8 secretions and PI3K/Akt, MAPK, and NF-*κ*B pathways activity induced by fMLP. Neutrophils were obtained from healthy volunteers. IL-8 was measured by ELISA, IL-8 mRNA by qPCR, and ROS production by luminol-amplified chemiluminescence, reduction of ferricytochrome c, and FACS. Intracellular pH changes were detected by spectrofluorescence. ERK1/2, p38 MAPK, and Akt phosphorylation were analysed by immunoblotting and NF-*κ*B was analysed by immunocytochemistry. Hydroxy-3-methoxyaceto-phenone (HMAP), diphenyleneiodonium (DPI), and siRNA Nox2 reduced the ROS and IL-8 release in neutrophils treated with fMLP. HMAP, DPI, and amiloride (a Na^+^/H^+^ exchanger inhibitor) inhibited the Akt phosphorylation and did not affect the p38 MAPK and ERK1/2 activity. DPI and HMAP reduced NF-*κ*B translocation induced by fMLP. We showed that IL-8 release induced by fMLP is dependent on NADPH oxidase, and ROS could play a redundant role in cell signalling, ultimately activating the PI3K/Akt and NF-*κ*B pathways in neutrophils.

## 1. Introduction

Polymorphonuclear neutrophils (PMNs) are the first line of defence against microorganisms and are the main cellular component in the acute inflammatory response.

Neutrophils are primarily activated by chemotactic factors such as fMLP [1] and PAF [2].

Both compounds bind to the neutrophil cell surface via specific seven transmembrane domain G-protein coupled receptors [3, 4] and induce the activation of MAPK, PI3K, and NF-*κ*B pathways in neutrophils [5, 6]. Notably, only fMLP has been described as a potent inducer of IL-8 in human neutrophils [7]. IL-8 is a member of the CXC chemokine family, relevant to the pathogenesis of several acute inflammatory processes and to the tissue damage associated with neutrophils [8]. Because PAF alone is not able to induce IL-8 production [9], the existence of differential mechanisms of cell signalling, essential for neutrophil IL-8 release induced by fMLP, seems likely. It is widely known that fMLP induces a significant increase in superoxide production via NADPH oxidase; in contrast, basal levels of superoxide are unaltered in PAF activated neutrophils [9, 10]. In fact, neutrophils produce a strong respiratory burst, resulting in the release of a diversity of radical oxygen species (ROS), during phagocytosis or following stimulation with a wide variety of agents [11]. ROS originate from the activation of NADPH oxidase, which is assembled at the plasma membrane. This reaction produces two superoxide anions (O_2_^−^) and 2H^+^. The H^+^ accumulation induces a transient intracellular acidification that activates several compensatory mechanisms such as Na^+^/H^+^ exchanger (NHE), H^+^ channels, and V-ATPase [12], which promote intracellular alkalinisation [5]. ROS have been proposed as signalling molecules that regulate diverse responses in neutrophils, including cytokine expression [13–15]. It has been described that the superoxide anion induces NF-*κ*B activation (I*κ*B*α* degradation and p65 NF-*κ*B translocation) and increases the expression of TNF*α* and macrophage inflammatory protein-2 in neutrophils [16]. However the role of ROS in cytokine expression is until now controversial in neutrophils. Human neutrophils from chronic granulomatous disease (CGD) that have genetic mutations in any of the components of the NADPH oxidase enzyme show an increase of IL-8 production induced by fMLP, suggesting that ROS reduce the IL-8 production in neutrophils [17]. Moreover, exposure of bone marrow-derived neutrophils to extracellular H_2_O_2_ diminished LPS induced activation of NF-B and expression of NF-B-dependent proinflammatory cytokines [18, 19].

In the present work, we present evidence that supports the role of NADPH oxidase in IL-8 release, the PI3K/Akt pathway, and NF-*κ*B activity in human neutrophils treated with fMLP.

## 2. Materials and Methods

### 2.1. Reagents

Platelet-activating factor (C-16), fMLP, actinomycin D, SN50, UO126, LY294002, and SB203580 were obtained from Calbiochem (La Jolla, CA, USA). Histochoice, andrographolide, 4-hydroxy-3-methoxyaceto-phenone (HMAP), diphenyleneiodonium (DPI), and monoclonal antibody against *β*-actin were purchased from Sigma-Aldrich (St. Louis, MO, USA). The Akt inhibitor (sc-394003) 1L-6-hydroxymethyl-chiro-inositol-2-[(R)-2-O-methyl-3-O-octadecylcarbonate] was purchased from Santa Cruz Biotechnology (Dallas, TX, USA). Hank's balanced salt solution (HBSS), Iscove's Modified Dulbecco's medium (IMDM) Penicillin-streptomycin, certified foetal bovine serum, hydroethidine (HE), BCECF-AM, and nitrocellulose membrane were purchased from Invitrogen (Grand Island, NY, USA). Monoclonal antibodies against phospho-ERK1/2, phospho-p38, phospho-Akt (ser473), Akt, p38, rabbit IgG-HRP, and mouse IgG-HRP were purchased from Cell Signalling (Beverly, MA, USA). Polyclonal antibodies against ERK1 (sc-94), p65 NF-*κ*B, Nox2 (sc-5827), gp91-phox siRNA, nonsilencing control siRNA, siRNA Transfection Reagent (sc-29528), and siRNA Transfection Medium (sc-36868) were purchased from Santa Cruz Biotechnology (Santa Cruz, CA, USA). Human IL-8 CytoSet Kit was purchased from Biosource International (Camarillo, CA, USA) and PE Mouse Anti-Human IL-8 (#554720) was purchased from BD Pharmingen. Proteases inhibitors were purchased from Roche Diagnostics (Indianapolis, IN, USA). Affinity Script Reverse Transcriptase and Brilliant II SYBR Green QPCR master mix were purchased from Stratagene (USA). SV Total RNA Isolation System was obtained from Promega (Madison, WI, USA). All other reagents and chemicals were purchased from Merck (Darmstadt, Germany).

### 2.2. Isolation of Neutrophils

Neutrophils were obtained from the fresh blood of healthy adult human volunteers in accordance with guidelines set by and with the approval of the Bioethical and Bio-Safety Committee of Universidad Austral de Chile. Blood was collected in ACD vacutainer tubes, and neutrophils were purified by discontinuous Percoll gradient centrifugation. Neutrophils were suspended in Hank's Balanced Salt Solution (HBSS) (5.33 mM KCl, 0.441 mM KH_2_PO_4_, 138 mM NaCl, 0.34 mM Na_2_HPO_4_, and 5.56 mM D-glucose). Purity and viability were greater than 95% as determined by May-Grünwald Giemsa staining and trypan blue exclusion, respectively.

### 2.3. Cell Viability

Neutrophils (5 × 10^4^/well) suspended in 100 *μ*L HBSS were incubated with 500 *μ*M HMAP, 10 *μ*M DPI, 100 and 500 *μ*M amiloride, 1 *μ*M UO126, 10 *μ*M LY294002, 10 *μ*M SB203580, 10 *μ*M AKT inhibitor, or vehicle (0.2% DMSO) for 30 min and stimulated with fMLP 100 nM for 4 h at 37°C. After that, we used the CellTiter-Glo Luminescent Cell Viability Assay according the manufacturer instruction (Promega, Madison, WI, USA).

### 2.4. Determination of IL-8 Release by ELISA

Neutrophils (2 × 10^6^) were incubated with HMAP, DPI, amiloride, UO126, LY294002, SB203580, or vehicle for 30 min and stimulated with PAF or fMLP for 4 h. Supernatants were collected, and IL-8 was determined according the manufacturer's instructions (IL-8 Kit, Biosource).

### 2.5. Determination of Intracellular IL-8 by Flow Cytometer

Neutrophils (1 × 10^6^) in HBSS were incubated with 500 *μ*M HMAP, 10 *μ*M DPI, 500 *μ*M amiloride, 1 *μ*M UO126, 10 *μ*M LY294002, 10 *μ*M Akt inhibitor, 10 *μ*M SB203580, or vehicle (0.2% DMSO) for 30 min and stimulated with fMLP for 4 h at 37°C. Afterward neutrophils were centrifuged (300 ×g) for 6 min. The cells were fixed using paraformaldehyde 4% in PBS by 10 minutes at room temperature. Then the cells were washed twice using 500 *μ*L of PBS. Afterward, the cells were permeabilized using 0.5% triton X-100 in PBS for 15 min and afterward washed twice with PBS. Then, neutrophils were incubated overnight at 4°C in 1% BSA-PBST (PBS-Tween 0.1%) containing PE Mouse Anti-Human IL-8 (1 : 100) or 0.25 *μ*g mouse isotype antibody (5415 from Cell Signaling). A sample lacking the primary antibody was included as a control. Cells incubated with isotype antibody were incubated with 1% BSA-PBST with 1 : 1000 PE goat anti-mouse Ig (#550589) from BD Pharmingen (CA, USA) for 2 h at room temperature in the dark. Finally, the cells were washed with PBS and suspended in 300 *μ*L of PBS, and they were assessed by flow cytometry FACSCanto II (BD, CA, USA) flow cytometer and analysed using FlowJo 7.6 software (FlowJo, OR, USA).

### 2.6. Real Time PCR of IL-8

Neutrophils (4 × 10^6^) were incubated with fMLP for 1 h, and then 10 *μ*M actinomycin D, 500 *μ*M HMAP, 10 *μ*M DPI, or vehicle was added for 1 or 3 h and total RNA was isolated. The RNA was treated with DNase and cDNA synthesis was made using 200 ng of total RNA. Real time PCR was performed using SYBR Green and primers of IL-8 and *β*-actin in MX3000P QPCR (Stratagene, USA) according to the conditions described elsewhere [20].

### 2.7. ROS Production

Luminol-amplified chemiluminescence: neutrophils (1 × 10^6^) were suspended in HBSS (250 *μ*L/well) with 50 *μ*M luminol in the presence or absence of inhibitors (HMAP, DPI, amiloride, UO126, LY294002, or SB203580) for 10 min. Cells were subsequently stimulated by the addition of different concentrations (1 nM–10 *μ*M) of PAF or fMLP, or 100 nM PAF or fMLP, and the emission of light was recorded by a luminometer at 37°C for 30 min.

Superoxide production: O_2_^−^ release was monitored spectrophotometrically at 37°C by measuring O_2_^−^ dismutase-inhibitable reduction of ferricytochrome c at 550 nm. Assays were performed in 96-well microtiter plates [21]. Control wells contained all components of the assay mixture plus O_2_^−^ dismutase (20 U/mL) to correct for ferricytochrome c reduction by agents other than O_2_^−^. Cells (3 × 10^5^) were suspended in HBSS (200 *μ*L/well), incubated with inhibitors for 10 min, and stimulated by the addition of 100 nM PAF or fMLP. Absorbance (optical density) at 550 nm was recorded by a microplate reader (Tecan, Sunrise). O_2_^−^ release was measured under conditions of linearity with respect to time and cell number, and O_2_^−^ release was expressed as nmol O_2_^−^/3 × 10^5^ PMNs [21]. Additionally, superoxide production was assessed by flow cytometry using the fluorescent probe hydroethidine (HE). HL-60/neutrophils cells were loaded with 10 *μ*M HE for 5 min at 37°C; then vehicle or fMLP was added and the superoxide production was measured at 10 min in FACSCanto II (BD, CA, USA) flow cytometer, with excitation at 488 nm and emission using a 610 nm absorbance long pass filter.

### 2.8. RNA Interference Assay

Small interfering RNA (siRNA) targeting human gp91-phox (Nox2) and a nonsilencing control RNA were used. HL-60 cells were differentiated to neutrophils using 1.3% DMSO in IMDM medium for 5 days. Differentiated HL-60 cells were transiently transfected with each siRNA in siRNA Transfection Medium according to the manufacturer's protocol. Approximately 48 h posttransfection total proteins were isolated and gp91-phox levels were detected by immunoblot. Also, cells were stimulated with fMLP and assessed for superoxide production by flow cytometry and IL-8 production by ELISA, according to the protocols described above.

### 2.9. Neutrophil Intracellular pH

PMNs (2 × 10^7^ cells/mL) were suspended in a pH 7.2 buffer (140 mM NaCl, 10 mM glucose, 1 mM KCl, 1 mM CaCl_2_, 1 mM MgCl_2_, and 20 mM HEPES) and incubated with BCECF-AM (2.5 *μ*M; Molecular Probes, Oregon, USA) for 30 min at 37°C. The cells were then washed twice and suspended at 4 × 10^6^ cells/mL. The 8 × 10^6^ BCECF-loaded neutrophils were incubated with either vehicle, HMAP, DPI, amiloride, UO126, LY294002, or SB203580 for 10 min, followed by exposure to fMLP or PAF. Fluorescence was measured in a thermoregulated spectrofluorometer (LS55 Perkin-Elmer) at 490 and 440 nm of excitation and 535 nm of emission. The solution was continuously stirred. Fluorescence was converted to pH units using nigericin methods of calibration [5].

### 2.10. Immunoblotting

Neutrophils (5 × 10^6^) were incubated with HMAP, DPI, amiloride, or vehicle for 30 min and then incubated with fMLP or PAF (100 nM) for 2 min. For ERK1/2, p38 MAPK, and Akt phosphorylation determinations, total protein extracts were prepared and resolved (50 *μ*g) by 12% SDS/PAGE. Immunoblotting was performed using monoclonal antibodies against phospho-ERK1/2 and total ERK1/2, phospho-p38 and total p38, and phospho-Akt (ser473) and total Akt [5]. Blots were developed with ECL. The primary antibodies were stripped, and each membrane was reprobed with an antibody recognising total nonphosphorylated protein. Reprobed signal was detected as described above.

### 2.11. Immunocytochemistry

Neutrophils were incubated with UO126, LY294002, SB203580, HMAP, DPI, SN50, or vehicle for 30 min and stimulated with fMLP for 30 min. Cytospin was performed, and cells were fixed with Histochoice for 10 min and washed three times with PBS. Cells were subsequently permeabilized with 0.3% Triton X-100 in PBS for 15 min and washed three times with PBS. Each cytospin spot was then incubated with blocking buffer (1% BSA, 5% nonfat milk, and PBS) for 1 hour followed by incubation with an antibody directed against p65 NF-*κ*B in blocking buffer overnight at room temperature. Cells were then washed three times with PBS and incubated with Alexa Fluor 488-conjugated goat anti-rabbit antibody (1 : 200) for 2 hours in the dark; nuclei were counterstained with propidium iodide. Cells were then washed with PBS, mounted with fluorescence medium, and examined by confocal microscopy (Fluoview 1000, Olympus). The Image ProPlus software 4.5.1 (Media Cybernetic, MD, USA) was used to measure nuclear or cytoplasm localization of p65 NF-*κ*B.

### 2.12. Statistical Analysis

Results are expressed as fold increase compared to control, percentage, or area under the curve (AUC) and reported as mean ± SE. An ANOVA was performed and Dunnett's multiple comparison test was applied using GRAPH PAD V 2.0. The level of significance used was 5%.

## 3. Results

### 3.1. fMLP Produces High Levels of IL-8 and ROS and Increases the Intracellular pH

**Figure 1:**
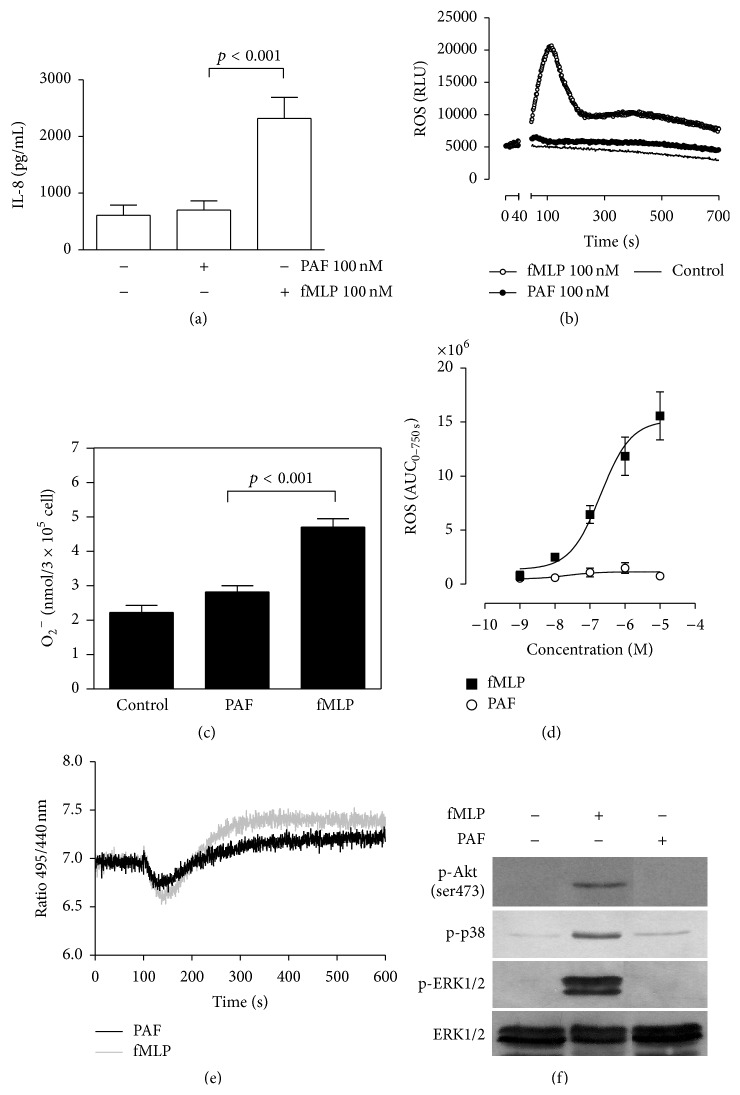
fMLP increases IL-8 release, ROS production, intracellular pH, and ERK1/2, p38 MAPK, and Akt phosphorylation. Human neutrophils were incubated with 100 nM fMLP or PAF for 4 h, and the IL-8 concentration in the supernatants was measured by ELISA (a). Neutrophils were incubated for 5 min at 37°C before fMLP or PAF was added. ROS production was monitored for 1200 s using a luminescence assay. RLU: relative luminescence unit (b), and superoxide production was measured following 30 min of incubation by a cytochrome c reduction assay (c). Curve dose response of ROS production in neutrophils stimulated with fMLP or PAF. AUC: area under curve for 700 s (AUC_700_) (d). BCECF-AM-loaded neutrophils were incubated for 5 min at 37°C, fMLP or PAF was added, and the signal was measured for 600 s in a spectrofluorometer (e). Neutrophils were incubated with fMLP or PAF for 2 min, and total protein was analysed by immunoblot with specific antibodies against the phosphorylated form of Akt, p38 MAPK, and ERK1/2. In this case the same membrane was used after stripping procedure for reprobed and total ERK1/2 antibody was used as a charge control (f). Mean ± SE, *n* = 3.

We determined the effects of fMLP and PAF on IL-8 release and ROS production as well as intracellular pH. The concentration of IL-8 was assessed by ELISA in supernatants of cells treated with each chemotactic factor for 4 hours. Only fMLP 1 induced an increase in IL-8 release compared to the basal control; IL-8 release induced by PAF was similar to the basal control (Figure 1(a)). Production of ROS was assessed by luminol-amplified chemiluminescence and reduction of ferricytochrome c, to measure total ROS and extracellular superoxide release, respectively. A rapid and significant increase in ROS and superoxide production was observed in fMLP treated neutrophils with maximum peak at 112 s (Figure 1(b)). Following this peak, ROS release decreased rapidly and a second peak of smaller intensity in some volunteers was observed. This minor peak was distinctive and unique for each individual volunteer. After 2 min of incubation, fMLP but not PAF stimulated an increase in superoxide release (Figure 1(c)). Only fMLP, in a dose-dependent manner, increased the ROS production (Figure 1(d)). NADPH oxidase activation also induces H^+^ release in the intracellular space, contributing to slight and transient neutrophil acidification [12]. This lower pH is spontaneously restored to normal by activation of the Na^+^/H^+^ exchanger, producing an increase of intracellular pH [5]. We assessed the changes in intracellular pH in fMLP or PAF treated neutrophils using a fluorescent BCECF-AM probe. We found that fMLP and PAF induce a similar intracellular acidification in neutrophils; however, fMLP triggered a large rebound increase in intracellular pH following the period of acidification (Figure 1(e)).

Previously, it had been demonstrated that MAPK and Akt phosphorylation are induced by fMLP and PAF in neutrophils [6, 22, 23]. Here we show that ERK1/2, p38 MAPK, and Akt phosphorylation are more intense when induced by fMLP compared to PAF (Figure 1(f)).

### 3.2. NADPH Oxidase Inhibition Reduces IL-8 Release by Neutrophils Treated with fMLP

**Figure 2:**
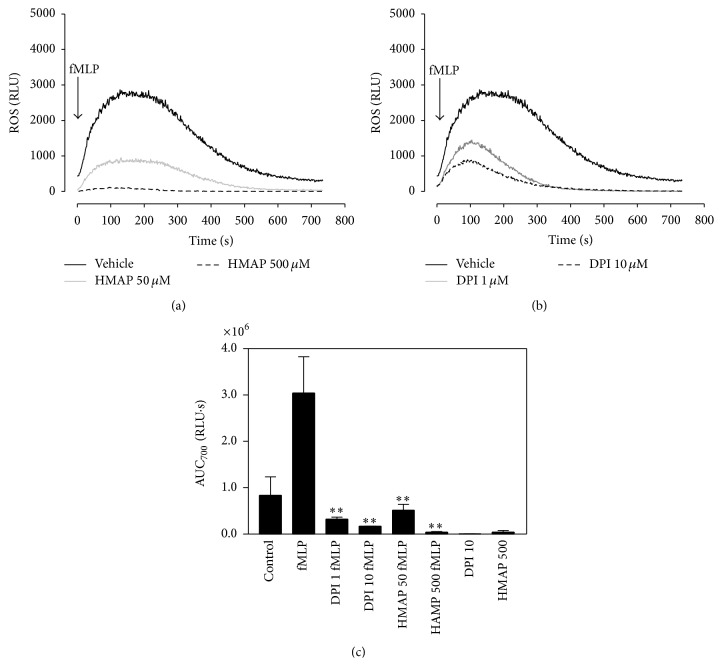
NADPH oxidase inhibition reduces ROS production. Neutrophils incubated with 50 or 500 *μ*M HMAP (a), 1 or 10 *μ*M DPI (b), or vehicle for 10 min were stimulated with 100 nM fMLP, and ROS production was detected by luminescence assay. In (c), the effect of NADPH oxidase inhibitors on AUC for 700 seconds (AUC_700_) of ROS production is shown. Mean ± SE; ^*∗∗*^ *p* < 0.01 compared to fMLP; *n* = 3.

ROS have been described as second messengers for the induction of cytokines [13]; however in neutrophils the role of ROS in the IL-8 release induced by fMLP is until now controversial. We assessed the role of ROS in IL-8 release by using DPI and HMAP, two known NADPH oxidase inhibitors. We observed that 1 and 10 *μ*M DPI as well as 50 and 500 *μ*M HMAP reduce, in a dose-dependent manner, the ROS production induced by fMLP in neutrophils as measured by luminol-amplified chemiluminescence (Figures 2(a)–2(c)).

**Figure 3:**
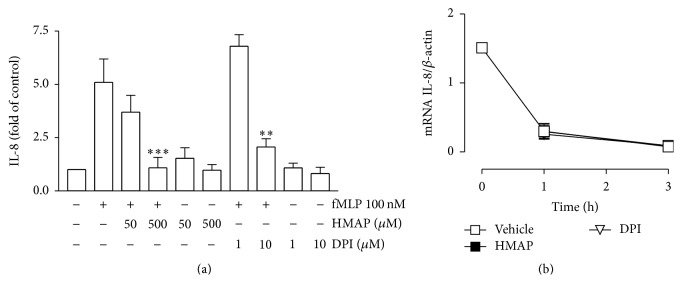
NADPH oxidase inhibition reduces IL-8 production. Neutrophils were treated with HMAP, DPI, or vehicle for 30 min and stimulated with fMLP for 4 h. IL-8 was measured in the supernatants by ELISA (a). Neutrophils were incubated with fMLP for 1 h, and then 10 *μ*M actinomycin D and 500 *μ*M HMAP, 10 *μ*M DPI, or vehicle were added and incubated for 1 or 3 h. Total RNA was isolated and cDNA synthesis and qPCR of IL-8 and *β*-actin were done (b). Mean ± SE; ^*∗∗*^ *p* < 0.01; ^*∗∗∗*^ *p* < 0.001 compared to fMLP; *n* = 3.

Subsequently, IL-8 release was measured in neutrophils treated with DPI or HMAP for 30 min and stimulated with fMLP for 4 hr. We observed that IL-8 release was reduced following treatment with 500 *μ*M HMAP and 10 *μ*M of DPI (Figure 3(a)). Additionally, we assessed the effect of HMAP or DPI on stability of IL-8 mRNA. Neutrophils were stimulated with fMLP for 1 h and then actinomycin D plus HMAP, DPI, or vehicle for 1 or 3 h was added. The total RNA was used for cDNA synthesis and real time PCR of IL-8 and *β*-actin.  Figure 3(b) shows that the treatments with HMAP or DPI did not modify the slope of IL-8/*β*-actin compared to the vehicle, indicating that NADPHox inhibitors did not affect the mRNA stability, suggesting an effect on IL-8 at transcriptional level. siRNA assay targeting human Nox2 was used to verify the effect of NADPHox inhibition on IL-8 release. HL-60 cells differentiated to neutrophils were used for transfection assay.

**Figure 4:**
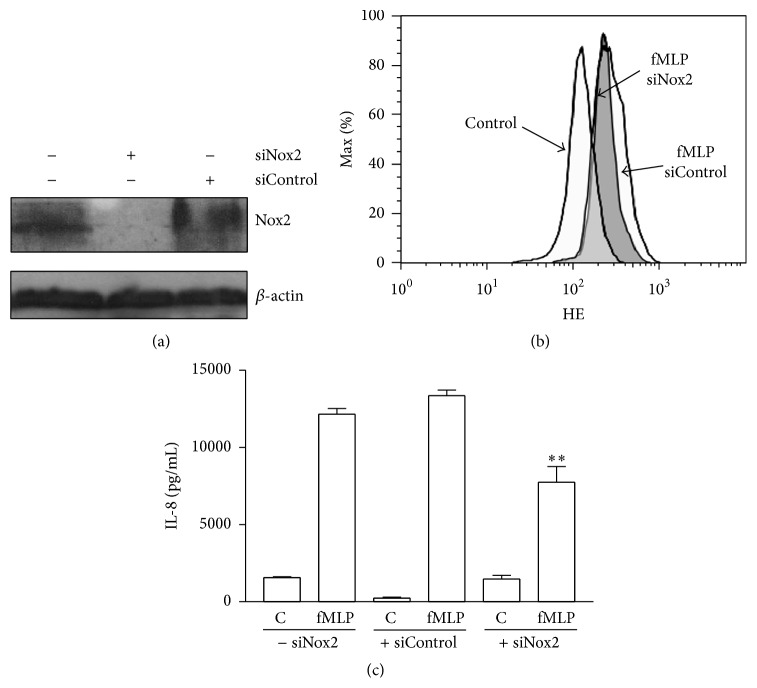
Nox2 siRNA interferes with ROS and IL-8 production in HL-60-derived neutrophilic cells. HL-60 cells were differentiated to neutrophils and transfected with Nox2 siRNA or control siRNA. (a) A representative immunoblot of Nox2 from cells untreated with siRNA or treated with unspecific siRNA (siControl) or specific siRNA (siNox2) is shown. As control *β*-actin was used. (b) HL-60/neutrophils transfected with siRNA siControl or Nox2 were loaded with HE and treated with vehicle (Control) or fMLP. The superoxide production was measured by flow cytometry. (c) HL-60/neutrophils untreated or treated with siRNA siControl or siNox2 were incubated with vehicle or fMLP for 4 h and IL-8 production in the supernatants by ELISA was analysed. Mean ± SE, ^*∗∗*^ *p* < 0.01 compared to the siControl cells treated with fMLP, *n* = 3.

Untransfected or transfected with siRNA Nox2 or siControl HL-60/neutrophils were used to determine Nox2 level, superoxide production, and IL-8 release. The transfection of siRNA Nox2 decreased the level of Nox2 compared to untransfected and siControl group (Figure 4(a)).

Also, a reduction of the superoxide production induced by fMLP in HL-60/neutrophils transfected with siRNA Nox2 compared to the untransfected or siControl group was observed (Figure 4(b)). Finally, we observed that the IL-8 release induced by fMLP was significantly reduced in HL-60/neutrophils transfected with siRNA Nox2 compared to untransfected or siControl transfected cells (Figure 4(c)).

### 3.3. HMAP and DPI Interfere with Intracellular pH Changes Induced by fMLP

**Figure 5:**
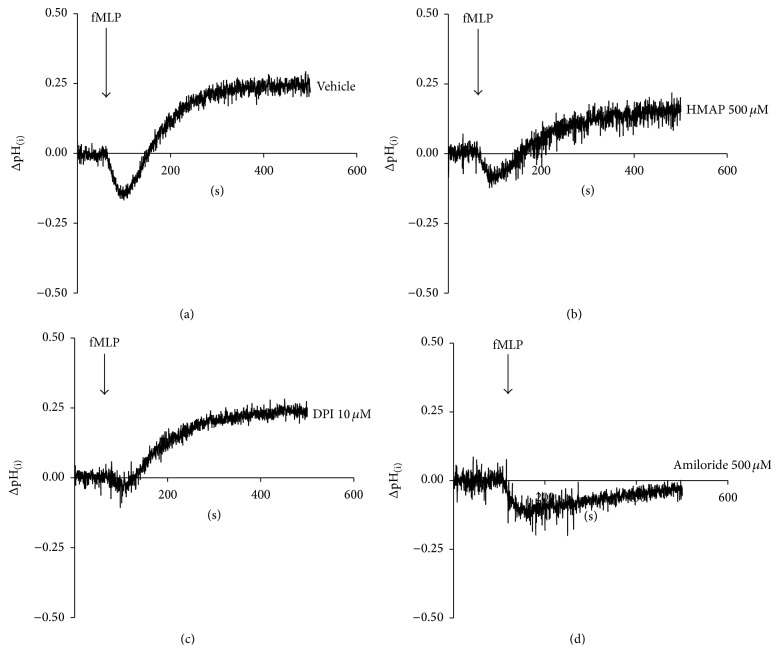
NADPH oxidase inhibition interferes with intracellular pH changes. BCECF-AM-loaded neutrophils were incubated with vehicle (a), 500 *μ*M HMAP (b), 10 *μ*M DPI (c), or 500 *μ*M amiloride (d) for 10 min. A basal level was measured before 100 nM fMLP was added, and the intracellular pH was recorded for 500 s.

Intracellular pH changes induced during fMLP activation could be associated with the respiratory burst [24]. The intracellular pH drop induced by chemoattractants is transient (Figure 5(a)); the recovery of intracellular pH is NHE dependent [5]. To assess the impact of NADPH oxidase inhibitors on intracellular pH changes, we assessed the effects of HMAP and DPI on intracellular pH changes induced by fMLP. We observed that HMAP partially and DPI completely inhibited intracellular acidification. In addition, HMAP, but not DPI, partially interfered with the intracellular alkalinisation induced by fMLP (Figures 5(b) and 5(c)). We observed that amiloride, a NHE inhibitor, strongly reduced the intracellular alkalinisation induced by fMLP (Figure 5(d)).

### 3.4. Amiloride Reduces Release of IL-8 and ROS Production in Neutrophils Treated with fMLP

**Figure 6:**
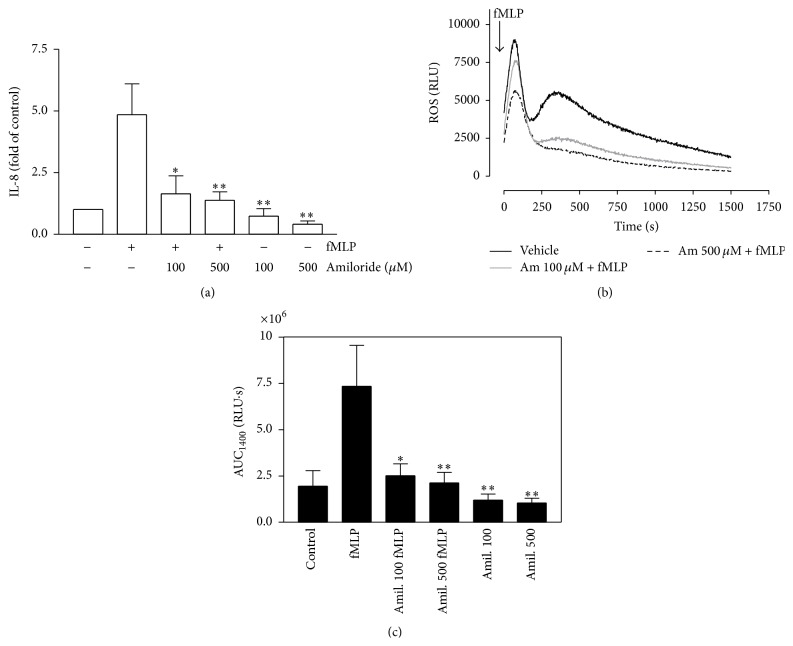
Amiloride reduces IL-8 and ROS production induced by fMLP. Neutrophils were incubated with vehicle or amiloride (100 or 500 *μ*M) for 30 min and stimulated with fMLP for 4 h. IL-8 production was analysed in the supernatants by ELISA (a). Neutrophils were incubated with vehicle or amiloride (100 or 500 *μ*M) for 10 min and then stimulated with 100 nM fMLP and the ROS production was detected by luminescence assay (b). In (c), the effect of amiloride on AUC for 1400 seconds (AUC_1400_) of ROS production is shown. Mean ± SE; ^*∗*^ *p* < 0.05; ^*∗∗*^ *p* < 0.01 compared to fMLP; *n* = 3.

It has been proposed that NHE is involved in IL-8 release [25]. To assess the role of NHE on ROS production and IL-8 release, we evaluated the effects of amiloride on human neutrophils treated with fMLP. Neutrophils were incubated with amiloride (an NHE inhibitor) for 30 min and stimulated with fMLP for 4 hr; IL-8 release was measured by ELISA. Amiloride (100 and 500 *μ*M) reduced IL-8 release in neutrophils stimulated by fMLP, which suggests a role for NHE in the release of this chemokine (Figure 6(a)). However, it appears that amiloride interferes with ROS production, and the reduction of IL-8 release is secondary (Figure 6(b)). This was evident when we measured the AUC of ROS production over 25 min (Figure 6(c)), supporting that amiloride also affects the ROS production in neutrophils activated by fMLP.

### 3.5. fMLP Induces IL-8 Release via MAPK, PI3K/Akt, and NF-*κ*B

**Figure 7:**
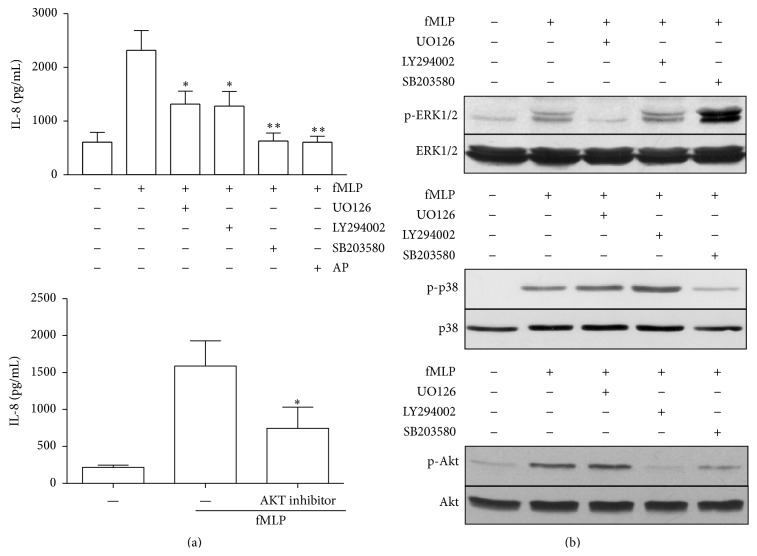
Effects of UO126, LY294002, Akt inhibitor, and SB203580 on IL-8 production and MAPK and Akt phosphorylation induced by fMLP. Neutrophils were incubated with vehicle, UO126 (1 *μ*M), LY294002 (10 *μ*M), SB203580 (10 *μ*M), andrographolide (AP) (50 *μ*M), or Akt inhibitor (10 *μ*M) for 30 min and stimulated with fMLP for 4 h. IL-8 production was analysed in the supernatants by ELISA (a). Neutrophils were incubated with vehicle, UO126 (1 *μ*M), LY294002 (10 *μ*M), or SB203580 (10 *μ*M) (b) for 30 min, stimulated with fMLP for 2 min, and analysed by immunoblot for ERK1/2, p38 MAPK, or Akt (Ser 473) phosphorylation. The blots were stripped and stained antibody specific to the unphosphorylated protein. Data presented are representative of three independent experiments. Mean ± SE; ^*∗*^ *p* < 0.05; ^*∗∗*^ *p* < 0.01 compared to fMLP; *n* = 3.

We analysed the signalling pathways that control IL-8 release. Neutrophils were pretreated with UO126 (MEK1/2 inhibitor), SB203580 (p38 MAPK inhibitor), or LY294002 (PI3K inhibitor) or with a specific Akt inhibitor for 30 min and stimulated with fMLP for 4 hr. A reduction in fMLP-induced IL-8 release was observed with all inhibitors analysed (Figure 7(a)). Furthermore, we demonstrated that andrographolide, a well-known NF-*κ*B inhibitor [26–28], reduces the IL-8 release induced by fMLP. Neutrophils were incubated with the vehicle or inhibitors (UO126, LY294002, or SB203580) for 30 min and stimulated with fMLP for 2 min before ERK1/2, p38 MAPK, or Akt phosphorylation were analysed by immunoblotting (Figure 7(b)). fMLP induced an increase in ERK1/2 phosphorylation, a response that was completely inhibited by UO126. The p38 MAPK phosphorylation induced by fMLP was inhibited by SB203580. Furthermore, the increase in Akt phosphorylation induced by fMLP was inhibited by LY294002. Notably, Akt phosphorylation was also reduced by SB203580, suggesting that p38 MAPK could be upstream of Akt; this observation is consistent with previous reports in other cells which have indicated a possible role for p38 MAPK in regulating Akt phosphorylation [29].

### 3.6. NADPH Oxidase, NHE, MAPK, and PI3K/Akt Inhibitors Increase the Intracellular IL-8 Level in fMLP Treated Cells

**Figure 8:**
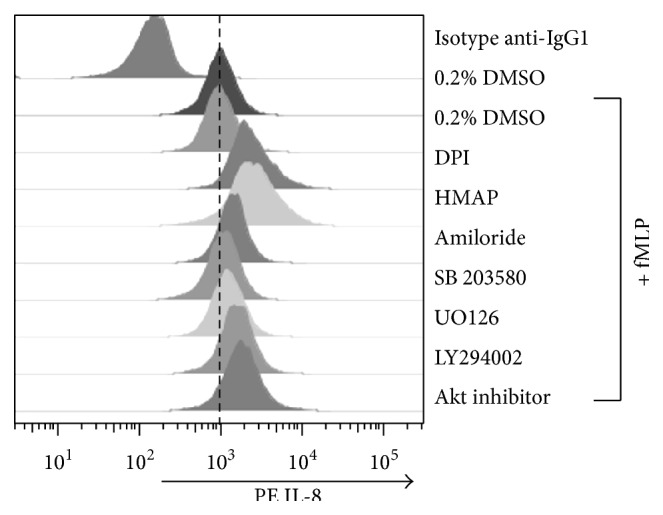
NAPDH oxidase, NHE, MAPK, and PI3K/Akt inhibitors increase the intracellular IL-8 level in fMLP treated cells. Neutrophils were incubated with vehicle, DPI (10 *μ*M), HMAP (500 *μ*M), amiloride (500 *μ*M), SB203580 (10 *μ*M), UO126 (1 *μ*M), LY294002 (10 *μ*M), or Akt inhibitor (10 *μ*M) for 30 min and stimulated with fMLP (100 nM) for 4 h. IL-8 intracellular content was measured by FACS. Data presented are representative of three independent experiments.

Because an interference of fMLP-induced IL-8 release was observed with the use of NADPH oxidase, NHE, MAPK, and PI3K/Akt inhibitors, a possible increase at intracellular level could be involved. To test this assumption we performed FACS experiments to assess the intracellular content of IL-8. We observed that DPI and HMAP increased the intracellular content of IL-8 in neutrophils stimulated with 100 nM fMLP. In a lesser extent, the inhibition of NHE or interference of PI3K/Akt, p38 MAPK, and ERK1/2 pathway also increased the intracellular level of this chemokine (Figure 8). Moreover, we discard a cytotoxic effect because none of these inhibitors affect the cellular viability (Supplemental Figure in Supplementary Material available online at http://dx.doi.org/10.1155/2015/120348).

### 3.7. NADPH Oxidase Inhibition Reduces Akt Phosphorylation

**Figure 9:**
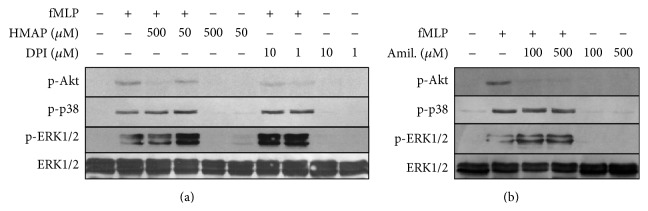
Effect of HMAP, DPI, and amiloride on MAPK and Akt phosphorylation induced by fMLP. Neutrophils were incubated with vehicle, HMAP, DPI (a), or amiloride (b) for 30 min, stimulated with fMLP for 2 min, and analysed by immunoblot for ERK1/2, p38 MAPK, or Akt (Ser 473) phosphorylation. The blots were stripped and stained antibody specific to the unphosphorylated protein. Data presented are representative of three independent experiments.

Because MAPK and PI3K/Akt participate in IL-8 release, we investigated whether NADPH oxidase activity has a role in ERK1/2, p38 MAPK, and Akt phosphorylation. Neutrophils were pretreated with DPI or HMAP for 30 min and subsequently stimulated with fMLP for 2 min; ERK1/2, p38 MAPK, and Akt phosphorylation were detected by immunoblotting. It was demonstrated that DPI and HMAP, in a dose-dependent manner, reduced Akt phosphorylation but did not affect ERK1/2 and p38 MAPK phosphorylation (Figure 9(a)). Also, we investigated the role of NHE in regulating MAPK and Akt phosphorylation. Amiloride strongly inhibited Akt phosphorylation but did not affect ERK1/2 or p38 MAPK phosphorylation, a pattern similar to that observed with NADPH oxidase inhibitors (Figure 9(b)).

### 3.8. fMLP Induces NF-*κ*B Activation via MAPK, PI3K/Akt, and NADPH Oxidase

**Figure 10:**
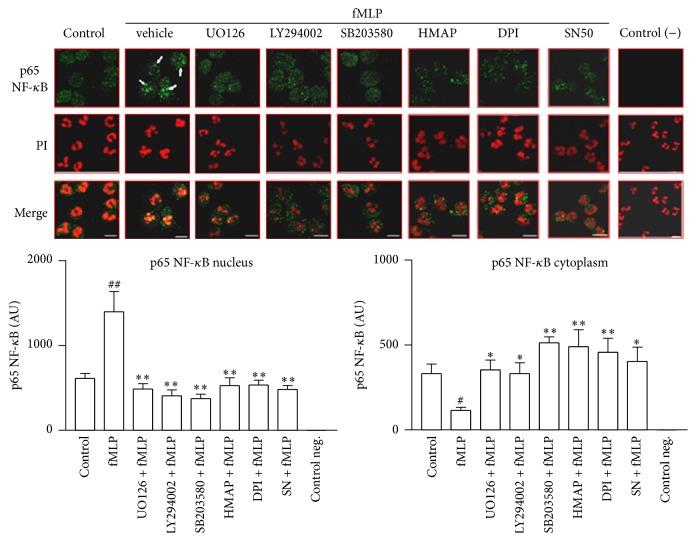
ERK1/2, PI3K, p38 MAPK, and NADPH inhibition reduce p65 NF-*κ*B translocation induced by fMLP. Neutrophils were incubated with vehicle, UO126 (1 *μ*M), LY294002 (10 *μ*M), SB203580 (10 *μ*M), HMAP (500 *μ*M), DPI (10 *μ*M), or SN50 (100 *μ*g/mL) for 30 min and then stimulated with fMLP for 30 min. The cells were fixed and analysed by immunocytochemistry with an antibody targeted against p65 NF-*κ*B. The nucleus was stained with propidium iodide (PI). The arrow shows the presence of p65 NF-*κ*B in nucleus. The negative control was prepared without anti-p65 NF-*κ*B. Bar: 10 *μ*m. Figure is representative of three independent experiments. Each bar represents mean ± SE of arbitrary units (AU) of fluorescence intensity of p65 NF-*κ*B, *n* = at least 5; ^*∗*^ *p* < 0.05; ^*∗∗*^ *p* < 0.01 compared to fMLP.

We evaluated NF-*κ*B activation using confocal microscopy to analyse the p65 NF-*κ*B translocation, a strongly expressed isoform in neutrophils [30]. In control cells, p65 NF-*κ*B preferentially showed a cytoplasmic distribution. However, when the cells were activated by fMLP, p65 NF-*κ*B was mainly localised in the nucleus (arrows in Figure 10); this localization was also visualised with the nuclear stain propidium iodide in merged images (Figure 10). UO126 markedly inhibited the nuclear translocation of p65 NF-*κ*B in PAF activated cells, confirming participation of the ERK1/2 pathway in NF-*κ*B activation. In cells activated with fMLP, preincubation with UO126, LY294002, and SB203580 significantly inhibited the p65 NF-*κ*B translocation, resulting in a distribution similar to that of the control cells (Figure 10). These results demonstrate the importance of the ERK1/2, PI3K, and p38 MAPK pathways in NF-*κ*B activation induced by fMLP. Additionally, we pretreated neutrophils with HMAP and DPI before stimulating the cells with fMLP. We observed that DPI and HMAP significantly interfered with NF-*κ*B translocation, suggesting that NADPH oxidase is involved in the activation of this pathway. SN50, a cell-permeable peptide carrying a functional domain, nuclear localization sequence, that inhibits nuclear translocation of NF-*κ*B/Rel complexes in intact cells, was used as control. SN50 significantly reduced the nuclear localization of p65 NF-*κ*B induced by fMLP (*p* < 0.01). This result was in concordance with an inhibition of p65 NF-*κ*B translocation from cytoplasmatic compartment.

## 4. Discussion

It has been reported that fMLP [7], but not PAF [9], is a potent inducer of IL-8 in human neutrophils. In fact, we demonstrated that fMLP, but not PAF, increases IL-8 release by neutrophils. Both chemoattractants induce a similar pattern of intracellular signaling pathways [5, 6, 31]. However, using two different approaches (luminol-chemiluminescence and reduction of cytochrome c) a clear difference between ROS production induced by fMLP and that induced by PAF in neutrophils was observed. It is widely known that PAF does not induce respiratory bursts and is considered mainly a priming stimulus in neutrophils [9]. There exist controversial antecedents in the role of ROS in neutrophils cytokine production. We hypothesised that fMLP induces IL-8 release via NADPH oxidase activity in neutrophils. We tested two NADPH oxidase inhibitors: HMAP, which reduces NADPH oxidase activity by competing with NADPH for the oxidase binding site [32], and DPI, which blocks flavin adenine dinucleotide binding to the oxidase [33]. HMAP and DPI, to a lesser extent, inhibited ROS production in neutrophils treated with fMLP. The observation that these compounds inhibit IL-8 release suggests that NADPH oxidase is involved in the secretion of this chemokine. We corroborate the role of NADPHox on IL-8 release induced by fMLP by using siRNA of Nox2 in HL-60/neutrophils. By the contrary, in neutrophils from chronic granulomatous disease that have genetic mutations in any of four components of the NADPH oxidase, fMLP increase the IL-8 neutrophil content [17]. These results could be explained by the different experimental conditions used. Because we measured the secretion of IL-8 but not total protein content, we propose that NADPH oxidase inhibition could be interfering with the release of IL-8, reducing the mobilization of a IL-8-containing organelle to the plasma membrane [34]. In fact, we observed that NADPH oxidase inhibitors increased the IL-8 at intracellular level in neutrophils treated with fMLP, suggesting interference in the release of this chemokine. In neutrophils, it has been observed that ROS are involved in IgE-induced IL-8 release [35]. Moreover, in neutrophils treated with LPS the IL-8 release was inhibited using OH radical scavenger [36].

Because NADPH oxidase activity is involved in intracellular acidification in neutrophils [24], we assessed the effects of the NADPH oxidase inhibitors on intracellular pH changes induced by fMLP. fMLP induced biphasic pH changes characterised by transient intracellular acidification followed by intracellular alkalinisation. HMAP partially affected the intracellular acidification and alkalinisation, and DPI only affected the intracellular acidification. Sustained intracellular acidification has been demonstrated to increase H_2_O_2_ but not O_2_^−^, which is explained by an increased rate of dismutation of O_2_^−^ at acidic intracellular pH [24]. Our results suggest that transient acidification alone is insufficient to increase IL-8; furthermore, PAF produced a similar intracellular pH pattern but did not induce IL-8 release in neutrophils. To assess the impact of intracellular acidification on IL-8 product, we subjected neutrophils to different concentrations of HCl. We found that intracellular acidification did not interfere with fMLP-induced chemokine secretion (data not shown). Additionally, we assess if intracellular alkalinisation via NHE could also be involved in IL-8 release. In monocytes and macrophages, LPS and IFN-*γ* via NHE promote the release of cytokines [25]. We found that amiloride inhibits fMLP-stimulated IL-8 secretion by neutrophils. However, amiloride also inhibited ROS production in neutrophils treated with fMLP, suggesting that NADPH oxidase could be involved in this IL-8 release. Moreover, PAF also activates NHE in neutrophils [5] but does not modify IL-8 basal levels or ROS production. Recently, it has been described that NHE inhibitors directly reduce mitochondrial function, thus preventing ROS production in cat myocardium induced by angiotensin II and endothelin-1 [37]. Therefore, we suggest that the effects of NHE inhibitors on neutrophils could be in part attributed to inhibition of ROS production.

The ROS could contribute to chemokine expression by acting as an intracellular second messenger, either directly or indirectly influencing the signalling pathways activated by chemoattractants such as fMLP. Our results show that NADPH oxidase inhibitors and amiloride reduced only the phosphorylation of Akt in neutrophils treated with fMLP, which indicate that MAPK pathways are not directly regulated by NADPH oxidase, suggesting the existence of a redundant effect of NADPH oxidase on PI3K/Akt pathways in neutrophils treated with fMLP, effect that could be cell and ligand specific. In support of this, LPS in macrophages, via NADPH oxidase, contribute to the phosphorylation of Akt but not p38 MAPK or ERK1/2 [38, 39]. However, angiotensin II potently induces phosphorylation of p38 MAPK and ERK1/2 in neutrophils, which is inhibited by NADPH oxidase inhibitors (e.g., DPI) and ROS scavengers [40]. In addition, HMAP reduces phosphorylation of p38 MAPK and ERK1/2 induced by *α*-IgE in neutrophils from sensitised allergic patients [41]; moreover, nonlethal concentrations of H_2_O_2_ have been demonstrated to activate p38 MAPK and ERK1/2 [42]. We demonstrated that SB203580, but not UO126, reduced Akt phosphorylation induced by fMLP in neutrophils. Therefore, our result supports that NADPH oxidase could contribute directly to the activation of PI3K in neutrophils stimulated by fMLP and would be necessary for IL-8 release [43]. In support of this, the inhibition of PI3K or Akt reduced the release of IL-8 induced by fMLP and increased the intracellular concentration of this chemokine. Our proposal suggests that NADPH oxidase participates in the activation of the PI3K pathway in neutrophils treated with fMLP. In fact, it recently has been described that Akt phosphorylation can be induced by ROS. Elevation of ROS can activate the PI3K/Akt pathway in TonB.210 cells with Bcr-Abl activated [44].

MAPK and PI3K/Akt pathways simultaneously play crucial roles in NF-*κ*B activation [45]; therefore, the contribution of NADPH oxidase on NF-*κ*B could be relevant in the IL-8 production induced with fMLP. HMAP and DPI reduced the p65 NF-*κ*B translocation in neutrophils. In addition, NF-*κ*B inhibition using andrographolide [27, 28, 45] reduced the IL-8 release in neutrophils treated with fMLP. In concordance with our results other reports show that NF-*κ*B controls the expression and release of IL-8 in LPS- or TNF-*α*-stimulated neutrophils [46].

In the present study, we additionally showed that fMLP induces p65 NF-*κ*B translocation in neutrophils and is upstream mediated by ERK1/2, PI3K, and p38 MAPK pathways. It has been suggested that there is a link between ERK1/2 and NF-*κ*B in neutrophils activated with group IB secretory phospholipase A2 [47]. Previously, other authors have indirectly suggested that ERK1/2 MAPK could participate in NF-*κ*B activation in human neutrophils, mainly because ERK1/2 is activated by TNF-*α*, a potent NF-*κ*B activator [48, 49]. A study showed that PI3K is upstream from NF-*κ*B activation in neutrophils activated with type 1 IFN and exerts an antiapoptotic effect [50]. The role of p38 MAPK in the regulation of NF-*κ*B activation in neutrophils is controversial. A report [46] concluded that p38 MAPK does not participate in NF-*κ*B binding in LPS-stimulated neutrophils; however, in the present study we demonstrate a role for p38 MAPK in activating this transcription factor following fMLP stimulation. Our results agree with those of other authors who described the contribution of p38 MAPK to NF-*κ*B activation in LPS-stimulated neutrophils [45]. The proteins downstream of p38 MAPK that control NF-*κ*B are unknown in neutrophils; however, we did not exclude Akt as a possible candidate since inhibition of PI3K and p38 MAPK reduces Akt phosphorylation induced by fMLP. A similar cross talk between p38 and PI3K/Akt has been previously suggested in different cells [29].

Based on these results, we conclude that fMLP can increase NADPH oxidase activity and ROS production, favouring the activation of PI3K/Akt and NF-*κ*B transduction pathways involved in the IL-8 release in neutrophils.

## Supplementary Material

In order to assess the effect of the inhibitors (DPI, HMAP, Amiloride, UO126, LY294002, SB203580 and Akt) on neutrophil viability, we used the Cell-Titer-Glo Luminescent Cell Viability Assay. We demonstrated that none inhibitors affected cellular viability, suggesting that the effect observed on the IL-8 release induced by fMLP, is not related with cytotoxicity.
