# Supplementary figures and images for: fMLP-Induced IL-8 Release Is Dependent on NADPH Oxidase in Human Neutrophils
Source: J Immunol Res. 2015 Nov 8;2015:120348. doi: 10.1155/2015/120348 (PMC4655063; doi:10.1155/2015/120348)

Supplemental Figure

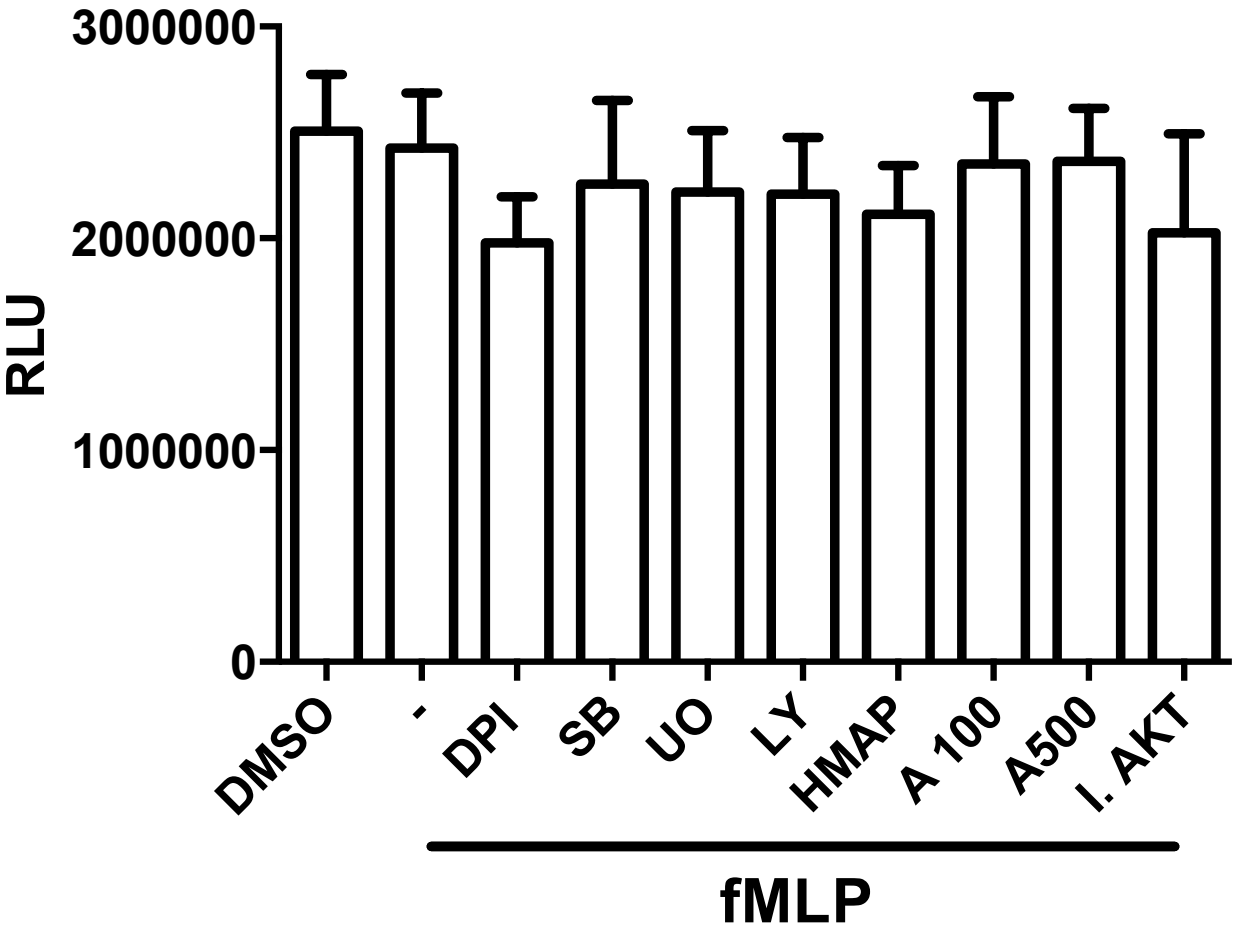

Supplement: Supplementary file 1 — In order to assess the effect of the inhibitors (DPI, HMAP, Amiloride, UO126, LY294002, SB203580 and Akt) on neutrophil viability, we used the Cell-Titer-Glo Luminescent Cell Viability Assay. We demonstrated that none inhibitors affected cellular viability, suggesting that the effect observed on the IL-8 release induced by fMLP, is not related with cytotoxicity. [file 120348.f1.pdf]
